# Leadless pacemaker as a solution for radiation exposure risk in a patient with lung cancer and a transvenous pacemaker

**DOI:** 10.1016/j.hrcr.2025.05.025

**Published:** 2025-06-03

**Authors:** Daiki Kumazawa, Takehiro Nomura, Keita Yoshiyama, Yosuke Mizuno, Kosuke Onodera, Kennosuke Yamashita

**Affiliations:** Heart Rhythm Center, Department of Cardiovascular Medicine, Sendai Kosei Hospital, Miyagi, Japan

**Keywords:** Leadless pacemaker, Transvenous pacemaker, Radiation therapy, Cardiac implantable electronic devices, Electromagnetic interference

**Key Teaching Points**

• Leadless pacemakers such as an Aveir^TM^ VR can be safely used in patients with cancer requiring radiotherapy, especially when the radiation field includes the site of a conventional transvenous pacemaker generator.
• An Aveir VR’s retrievability, low battery consumption during frequent device checks, and longer battery longevity make it particularly suitable for patients undergoing radiation therapy.
• Delayed wound healing at the generator removal site should be carefully monitored, especially when transvenous leads are left in place, given that they may increase the localized radiation dose.
• Compared with a Micra^TM^, an Aveir VR lacks atrioventricular synchrony and remote monitoring, but offers the advantage of potential retrieval, repositioning, and future dual-chamber system (Aveir DR) upgradeability.

## Introduction

Radiation therapy can cause a malfunction of cardiac implantable electronic devices due to electromagnetic interference or an overcurrent. Although rare, such malfunctions may lead to life-threatening outcomes. One reported case described inappropriate ventricular pacing caused by software errors in an implantable cardioverter defibrillator, which led to ventricular tachycardia.^1^ Another case reported rapid atrial pacing due to pacemaker software errors, resulting in hypotension.^2^ In the latter case, the device failed to accept external setting changes, and the patient's sinus rhythm was restored only after disconnecting the lead. For cancer patients with bradyarrhythmias, a Micra^TM^ (Medtronic, Minneapolis, MN) leadless pacemaker (LP) can serve as a viable alternative to avoid direct radiation exposure to the pacemaker generator and malfunctions.^3^ However, to our knowledge, no previous reports have described radiation therapy in patients implanted with an Aveir^TM^ VR LP (Abbott, Chicago, IL). We herein report on 2 cases in which an Aveir was implanted and a previously implanted pacemaker generator was removed prior to radiotherapy for lung cancer. This approach was taken due to the proximity of the generator to the pulmonary tumors, which posed a malfunction risk during radiation therapy. After the Aveir implantation, radiation therapy was successfully performed.

## Case report

**Figure 1:**
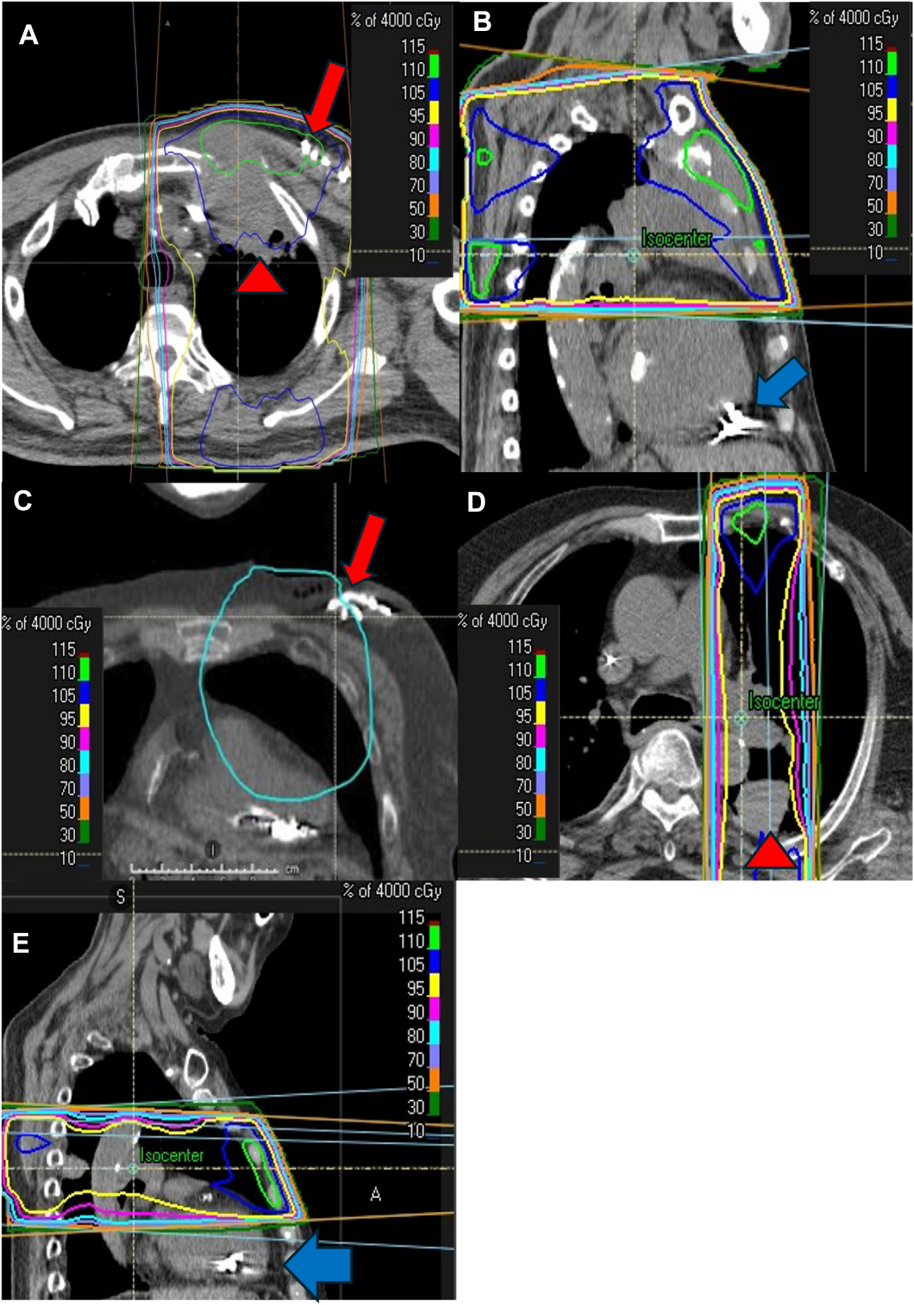
Radiotherapy planning images showing isodose lines in the axial, sagittal, and coronal planes. **A, B**: Images from the first case. **A:** Axial CT image demonstrating the lung tumor and the previously implanted pacemaker. The low-density area in the left upper lung (red arrowhead) indicates a squamous cell carcinoma requiring radiation therapy. The radiation field encompasses the pacemaker generator (red arrow), indicating a direct radiation exposure of approximately 40 Gy. **B:** Sagittal CT image showing the Aveir VR implanted on the right ventricular apical septum (blue arrow), positioned outside the radiation field to avoid direct exposure. **C, D, E:** Images from the second case. **C:** Coronal CT image acquired during expiration shows that the radiation field includes the pacemaker generator (red arrow), indicating a direct radiation exposure of approximately 30 Gy. **D:** Axial CT image showing a large-cell neuroendocrine carcinoma in the left lower lobe (red arrowhead). **E:** Sagittal CT image showing the Aveir VR located in the right ventricular apical septum (blue arrow), placed to avoid direct radiation exposure. CT = computed tomography.

The first case involved an 83-year-old male with complete atrioventricular block who had received a dual-chamber pacemaker (Evity 8 DR-T, BIOTRONIK SE & Co. KG, Berlin, Germany), consisting of a conventional atrial pacing lead (Solia S53, BIOTRONIK SE & Co. KG) and a ventricular pacing lead (Solia S60, BIOTRONIK SE & Co. KG), implanted in the left pectoral region 4 years prior, with a 100% ventricular pacing rate. The left ventricular ejection fraction was preserved, and was 82%. The patient presented with a stage III–B (cT4N2M0) squamous cell carcinoma in the left lung (92 mm diameter, in left S1–3 segments), and a plan was made to proceed with chemoradiotherapy (Figure 1A). However, it was anticipated that the radiation field would include the pacemaker generator. As a result of a discussion between the radiation oncologists and cardiologists, the generator was removed, and we implanted an Aveir VR before radiotherapy. The Aveir was positioned outside the radiation field (Figure 1B), and the radiation therapy began 2 weeks after the implantation. The patient successfully completed all planned radiation therapy sessions without any radiation interference or complications. Following radiation therapy, the patient developed radiation dermatitis (grade 1) and radiation esophagitis (grade 1), both of which were mild. The estimated radiation dose was 60 Gy/30 fractions, with an estimated average dose of the Aveir of 0.86 Gy and maximum dose of 1.39 Gy. Daily device checks before and after the radiation revealed no abnormalities, including any inappropriate pacing due to software errors. After the radiation therapy, the electrical parameters exhibited no calculated sensed R waves due to complete atrioventricular block, a pacing impedance of 510 Ω, with a pacing threshold of 0.5 V at a pulse width of 0.4 ms, which did not exhibit any deterioration as compared to the pre-radiation therapy values of a sensed R wave of 6.5 mV due to a transvenous pacemaker, pacing impedance of 960 Ω, and pacing threshold of 1.0 V at 0.4 ms. The QRS duration during pacing remained comparable (160 ms before and 164 ms after the Aveir VR implantation), suggesting stable ventricular conduction. The patient was scheduled for a device check 2 months after completing radiation therapy; there were no significant changes in device parameters at that time. Unfortunately, 6 months after completing treatment, the patient died of a myocardial infarction.

The second case involved an 83-year-old male with sick sinus syndrome who had received a single-chamber pacemaker (Attesta SR MRI SureScan, Medtronic) with a conventional ventricular pacing lead (Capsure Fix Novus 5076–58, Medtronic), implanted in the left pectoral region 14 years prior (Figure 1C). The left ventricular ejection fraction was preserved, and was 71%. Chemoradiotherapy was planned for a stage III–A (cT3N1M0) large-cell neuroendocrine carcinoma of the lung (42 mm diameter, in left S6 segment), and an Aveir VR was implanted the day before removing the generator (Figure 1D). Radiation therapy was initiated the day after the implantation of the Aveir, which had been placed outside the planned radiation field (Figure 1E). The estimated radiation dose was 60 Gy/30 fractions, with an estimated average dose of the Aveir of 1.7 Gy and maximum dose of 2.7 Gy. The device was checked before and after each day of irradiation, and no abnormalities, including inappropriate pacing due to software errors, were found. After the radiation therapy, the electrical parameters exhibited a sensed R wave of 11.0 mV, pacing impedance of 410 Ω, with a pacing threshold of 0.5 V at a pulse width of 0.4 ms, which did not exhibit any deterioration as compared to the pre-radiation therapy values of a sensed R wave of 10.0 mV, pacing impedance of 460 Ω, and pacing threshold of 0.5 V at 0.4 ms. Approximately one year has passed since the completion of radiation therapy, and no pacemaker malfunction has been observed. The patient developed radiation pneumonitis (grade 2) 4 months after completing treatment, but the condition improved with steroids, and home oxygen therapy was not required. The patient will continue to undergo annual follow-up.

## Discussion

In these cases, the pacemaker generators were removed while the leads were retained in situ, and the Aveir VR was implanted prior to the radiation therapy. In the first case, the generator of the transvenous system was removed after the Aveir VR was implanted during the same surgery. In the second case, the generator was removed the day after the Aveir VR was implanted. In these cases, the generator was removed early at the request of the radiation oncologist to facilitate an earlier initiation of radiation therapy; however, a brief delay might have been acceptable. In the end, no malfunctions of the Aveir VR were observed, indicating that radiation therapy is safe in patients implanted with it, similar to the previously reported cases involving a Micra implantation.^3^ In cancer patients with pacemakers undergoing radiation therapy, avoiding radiation exposure to the generator is critical. The options include either complete removal of the existing system or removal of the generator only. For the new pacemaker system, implantation of a transvenous pacemaker on the contralateral side or an LP can be considered. Regarding the necessity of generator removal, in the context of planned radiation therapy for cancer patients, retaining the generator is not recommended, as it can elevate the risk of radiation-induced skin injury due to scattered radiation. In these 2 cases, we considered either the complete device removal or implantation of a new transvenous pacemaker on the contralateral side. However, these patients had a preserved cardiac function and did not require an upgrade to cardiac resynchronization therapy. Moreover, considering their advanced age, a lead extraction posed a risk of complications, and the increased number of transvenous leads could have resulted in adverse outcomes such as tricuspid regurgitation or a superior vena cava obstruction. Compared to conventional transvenous pacemakers, an LP is associated with fewer short- and mid-term complications,^4^ potentially avoiding interruptions in cancer treatment due to device-related issues. In addition, the use of an LP is expected to enable future radiation therapy, even in cases of a pulmonary metastasis on the contralateral side. Consequently, the decision was made to remove only the generator and promptly implant the Aveir VR, without the complete removal of the device system or implantation of a new transvenous pacemaker on the contralateral side. After this procedure, careful attention had to be paid to delayed wound healing at the generator removal site. This healing delay is primarily attributable to radiation-induced skin injury. Furthermore, if transvenous leads are retained, scattered radiation from the leads may increase the local radiation dose to the wound site, thereby exacerbating the delay in wound healing. Therefore, although meticulous management was required in the present cases, the wounds healed without any complications.

As in the present cases, the Aveir VR could be an effective alternative for patients with bradyarrhythmias who require radiation therapy. The advantages and disadvantages of both the Aveir and Micra should be carefully considered when choosing between them.

The Micra has remote monitoring capabilities. Generally, adverse effects on pacemakers from radiation therapy are thought to occur during radiation exposure,^5^ and few cases have been reported of pacemaker malfunction in the remote period after radiation therapy. However, for LPs, which have limited clinical experience, continuous monitoring and management are essential to address any potential delayed complications. This makes the Micra a reasonable choice. On the other hand, the Aveir offers a removal tool, enabling repositioning and replacement if it is exposed to radiation due to disease progression. This feature expands treatment options, making the Aveir a beneficial alternative. Furthermore, the Aveir VR has the advantage of a longer battery longevity compared to the Micra.^6^ Additionally, the Aveir’s unique method of interrogation consumes less power, making it possible to minimize the battery consumption during interrogations of patients undergoing radiation therapy, who require frequent LP checks. Although the Aveir VR does not support atrioventricular synchronization, the future introduction of the Aveir DR and the addition of the Aveir AR are expected to address this limitation.^7^

## Conclusion

In conclusion, switching to the Aveir VR prior to radiotherapy may be a safe and effective strategy for cancer patients with bradyarrhythmias, especially when the radiation field includes the site of a conventional pacemaker generator. This approach minimizes the risk of device malfunction and may support uninterrupted oncologic treatment. Further studies with larger cohorts are warranted to confirm the long-term safety and clinical outcomes of radiation therapy. Overall, the implantation of the Aveir VR may represent a valuable treatment strategy for patients requiring radiation therapy near the pacemaker generator site, offering both safety and procedural feasibility.

## Disclosures

K.Y. received speaker honoraria and lecture fees from Abbott Medical Japan, Biosense Webster, Daiichi Sankyo, and Medtronic. The other authors have no conflict of interest.
